# Heterogeneity and chimerism of endothelial cells revealed by single-cell transcriptome in orthotopic liver tumors

**DOI:** 10.1007/s10456-020-09727-9

**Published:** 2020-05-21

**Authors:** Qi Zhao, Maria del Pilar Molina-Portela, Asma Parveen, Alexander Adler, Christina Adler, Hock E, Wei Wang, Min Ni, Yi Wei, Gurinder Atwal, Markus Mohrs, Gavin Thurston, Alexandra Eichten

**Affiliations:** Regeneron Pharmaceuticals, 777 Old Saw Mill River Rd, Tarrytown, NY 10591 USA

**Keywords:** Single cell transcriptome, Endothelial cell heterogeneity, Kupffer cells, Adjacent normal tissue, Liver tumor endothelial cells

## Abstract

The liver is a common host organ for cancer, either through lesions that arise in liver epithelial cells [e.g., hepatocellular carcinoma (HCC)] or as a site of metastasis by tumors arising in other organs (e.g., colorectal cancer). However, the changes that occur in liver stromal cells in response to cancer have not been fully characterized, nor has it been determined whether the different sources of liver cancer induce distinct stromal changes. Here, we performed single-cell profiling of liver stromal cells from mouse models of induced spontaneous liver cancer or implanted colorectal liver metastases, with a focus on tumor endothelial cells (ECs). While ECs in liver tissue adjacent to cancerous lesions (so-called adjacent normal) corresponded to liver zonation phenotypes, their transcriptomes were also clearly altered by the presence of a tumor. In comparison, tumor EC transcriptomes show stronger similarities to venous than sinusoidal ECs. Further, tumor ECs, independent of tumor origin, formed distinct clusters displaying conserved “tip-like” or “stalk-like” characteristics, similar to ECs from subcutaneous tumors. However, they also carried liver-specific signatures found in normal liver ECs, suggesting an influence of the host organ on tumor ECs. Our results document gene expression signatures in ECs in liver cancer and show that the host organ, and not the site of tumor origin (liver versus colorectal), is a primary determinant of EC phenotype. In addition, primarily in tumors, we further defined a cluster of chimeric cells that expressed both myeloid and endothelial cell markers and might play a role in tumor angiogenesis.

**Electronic supplementary material:**

The online version of this article (10.1007/s10456-020-09727-9) contains supplementary material, which is available to authorized users.

## Introduction

The liver is a common host organ for cancer. Tumors in the liver occur either spontaneously through lesions that arise in liver epithelial cells (e.g., hepatocellular and cholangiocarcinomas), or by metastatic spread from primary tumors in other organs (e.g., colorectal cancer). Even though tumors in the liver are quite common, the changes that occur in liver stromal cells in response to tumors have not been well characterized. Neither has it been determined whether the different cancer types in the liver induce distinct stromal changes.

Studies using single cell transcriptome profiling in various tumor types have revealed extensive heterogeneity in tumor cells and tumor-associated stromal cells, including endothelial cells (ECs), fibroblasts, smooth muscle cells and immune cells [1–3]. The extent and composition of this heterogeneity is thought to be shaped by interactions with other cells in the tumor mass [1, 2], and could thus be affected by various factors beyond the tumor type. For example, it was shown that malignant cells primarily clustered based on their original tumor identity, whereas non-malignant stromal cells in the tumor mass predominantly clustered based on their cell type [4, 5], suggesting shared properties within stromal cells across tumor samples of the same cancer type. In addition, a comprehensive analysis of bulk RNA profiling from tumor, adjacent normal, and naïve (non-tumor-bearing) tissue, using the GTEx and TCGA datasets, revealed that so-called normal adjacent tissue presents an intermediate state between naïve/healthy and tumor [6], suggesting a strong effect of a tumor on the non-tumor portion of the organ.

We previously employed single-cell transcriptome profiling to characterize tumor angiogenesis and EC heterogeneity compared to normal ECs [1]. Our findings better defined the heterogeneity of ECs in tumors, but these studies were limited to subcutaneous (s.c.) tumors, and thus did not provide information on how the host organ could affect the molecular signature. Another limitation of those studies was that the choice of normal tissue (heart) was not the naïve normal or adjacent-normal tissue corresponding to the subcutaneous space where the tumors were grown.

Here we performed single cell profiling of murine liver stromal cells in response to both an inducible liver cancer and to colorectal cancer metastases, with a focus on tumor ECs. In normal liver, we found distinct transcriptomes in EC subpopulations according to liver zonation. In liver tissue adjacent to cancerous lesions (so-called adjacent normal), the liver EC transcriptome was influenced by the tumor. Intrahepatic tumor ECs from inducible liver cancer and colorectal cancer metastases showed a high gene signature overlap with portal vein ECs and displayed gene signatures that resembled those found previously in subcutaneous tumors, including markers for “tip-like” and “stalk-like” endothelial cells. However, they also carried liver-specific signatures found in normal liver ECs, suggesting a strong influence of the host organ on tumor ECs. Additionally, we detected a cluster of chimeric cells, primarily in tumors, that expressed both myeloid and endothelial cell markers.

## Results

### Single cell profiling of endothelial cells (ECs) from normal liver

**Fig. 1:**
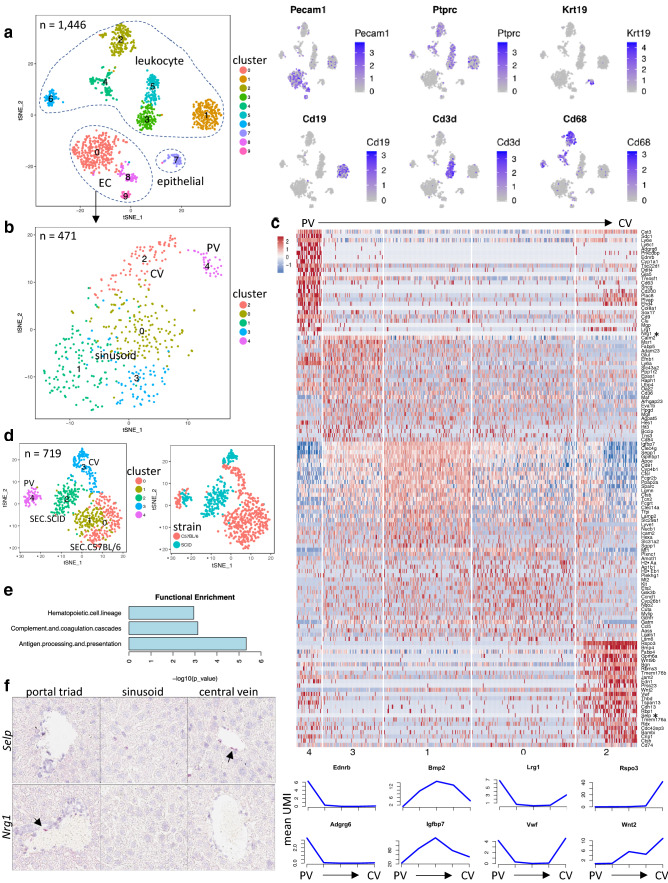
Molecular heterogeneity of endothelial cells (ECs) in normal liver. **a** Left, t-SNE plot showing clusters identified from 1,446 cells collected from normal C57BL/6 mouse liver tissue. Right, expression of cell-type marker genes in the t-SNE plot. Expression level UMI in natural log scale: blue, high; gray, low. **b** Further clustering of ECs in the C57BL/6 normal liver. Five subpopulations detected including sinusoid (clusters 0, 1 and 3), central vein (CV, cluster 2) and portal vein (PV, cluster 4). **c** Upper, heatmap of EC subpopulation-specific genes. Liver EC subpopulations are arranged in a zonation order from PV to CV based on a few well-established zonation markers such as *Rspo3*, *Wnt2* and *Msr1* etc. The numbers below in the heatmap correspond to cluster numbers as in 1b. Asterisk, two genes chosen to be validated by RNAScope (see 1f). Lower, expression profile (mean UMI within the cluster) of selected zonation genes by line plot linking points representing five clusters in the same order as in the heatmap above. **d** t-SNE plot of combined normal liver ECs from C57BL/6 and SCID mice. Left, colored by identified clusters; Right, colored by strain background. **e** Functional enrichment of genes preferentially expressed in liver sinusoid of C57BL/6 compared to SCID mice. **f** RNAScope validation of a novel central vein marker (*Selp*) and a novel portal vein marker (*Nrg1*) in normal liver from SCID mice. CV, central vein; PV, portal vein; SEC, sinusoid endothelial cells

To gain baseline information on liver ECs, we performed single cell transcriptome profiling on naïve normal liver from immune-competent C57BL/6 and immune-deficient SCID (C.B-17 scid) mice. Our single cell data were derived from multiple 10× Genomics sequencing runs. We benchmarked and observed that batch effect was minimal and rarely impacted cell clustering result (see method section for more info on minimization and monitoring of batch effect). A total of 1446 cells from normal C57BL/6 mouse livers were mapped into ten clusters representative of various leukocyte cell types (cluster 1–6), epithelial cells (cluster 7) and ECs (cluster 0, 8–9) (Fig. 1a and Supplementary Fig. 1a–c). Further, re-clustering of 471 ECs identified five subpopulations within the EC population (Fig. 1b and Supplemental Table 1). Consistent with the structure of the liver vasculature, we were able to annotate these subpopulations with consensus markers as central vein (CV) ECs (cluster 2, using *Rspo3* as a marker) [7], sinusoidal ECs (SEC) (cluster 0, 1 and 3, using *Clec4g* as a marker) [8, 9] and portal vein (PV) ECs (cluster 4) (Fig. 1c). Using gene expression patterns across subpopulations together with a few well-established EC zonation markers such as *Rspo3* and *Bmp2*, we were able to spatially assign the three SEC clusters along the PV to CV axis, representing EC zonation (Fig. 1c). Analysis of single cells collected from normal livers of naïve SCID mice aligned with our findings from C57BL/6 mice regarding EC subpopulations and subpopulation-specific marker genes (Supplementary Fig. 1d–e).

To assess whether there are mouse strain-specific differences in naïve liver ECs, we performed cell clustering on combined ECs from C57BL/6 and SCID livers (Fig. 1d). ECs from C57BL/6 and SCID mice aggregated into the same CV or PV clusters (cluster 3 and 4, respectively) (Fig. 1d and Supplementary Fig. 2a). However, within each CV or PV cluster, ECs segregated by strain. Sinusoidal ECs were again classified into three clusters, which also sub-segregated by strain: one cluster (cluster 2) was SCID-derived, whereas the other two clusters (cluster 0, 1) were from C57BL/6 mice. Strain-specific genes were found in liver ECs (Supplemental Table 1 and Supplementary Fig. 2b) with more differentially detected genes in SEC than PV or CV, due to higher SEC cell counts. Functional enrichment analyses of these differential genes showed that genes highly expressed in SECs of C57BL/6 mice were significantly associated with immune-related pathways, such as antigen presentation and processing, complement cascade and hematopoietic cell lineage (Fig. 1e). For example, expression of genes involved in MHC class II antigen presentation including *Cd74*, *H2-Ab1*, *Ctsb*, and *Lgmn* were significantly higher in SECs derived from immune-competent mice (C57BL/6) compared to SCIDs. On the other hand, genes more highly expressed in the sinusoidal ECs of SCID mice showed enrichment in ribosomal genes and oxidative phosphorylation. These findings suggested that the immune status may contribute to the transcriptional profile of liver ECs.

To benchmark the EC subpopulation and zonation genes derived from this study, we compared the results to recently published human [10] and mouse [8] studies. While the conservation of zonation genes was limited between mouse and human liver ECs, a number of reported mouse liver EC zonation profiles [8] did exhibit similar zonation patterns in our dataset, such as *Sox17* and *Dll4* as periportal EC markers (Supplementary Fig. 2c). However, the expression patterns of many reported zonation genes [8] were not reproduced in our current study. To further validate our zonation findings, we performed RNAScope for validation of marker expression in the respective specialized liver vessel structures. As expected *Rspo3* was highly expressed in the CV [7] (Supplementary Fig. 2d). Additionally, we were able to confirm *Selp* as another CV-enriched gene and *Nrg1* as a PV-specific gene (Fig. 1f).

**Fig. 2:**
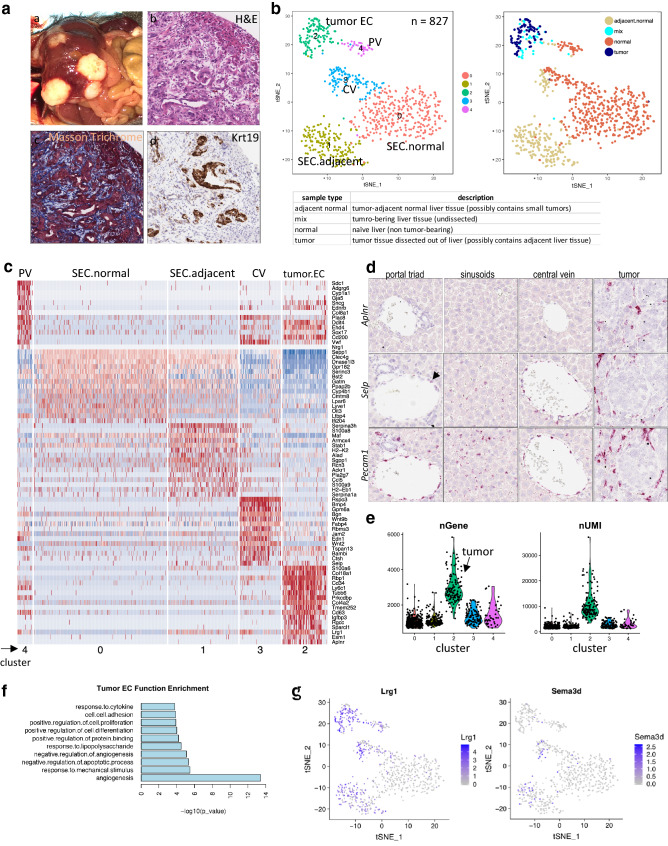
Distinct tumor EC and adjacent normal EC subpopulations in tumor-bearing liver tissues. **a** Characterization of HDD-induced intrahepatic tumors. (**a**) Gross image of liver harboring multiple HDD-induced tumors. Histological features of HDD-induced tumors by H&E (**b**), Masson Trichrome (**c**) and keratin 19 (*Krt19*) RNAScope (**d**) at × 20 magnification. **b** t-SNE plot of combined ECs collected from normal liver, dissected tumor, tumor-adjacent normal and undissected tumor-bearing liver (tumor and adjacent normal combined) tissue from C57BL/6 mice. Left, single cells colored by identified clusters; Right, cells colored by sample type. Bottom, nomenclature of sample types. **c** Heatmap of top 15 EC cluster-specific genes. Clusters (corresponding to the t-SNE plot in 2b) were annotated based on both known EC marker genes and sample type. **d** RNAScope on intrahepatic tumors and adjacent normal liver tissues of SCID mice showing tumor-specific expression of *Aplnr* and upregulation of *Selp* in sinusoid and PV (see naïve normal tissue expression in Fig. 1f for comparison). *Pecam1* was used as pan-EC marker. **e**. Gene and UMI counts across clusters (corresponding to t-SNE plot in 2b). **f** Functional enrichment (GO terms) of genes preferentially expressed (upregulated) in tumor ECs compared to non-tumor liver ECs. **g** Feature plot showing upregulation of *Lrg1* and *Sema3d* in adjacent normal ECs compared to naïve ECs (corresponding to EC cluster t-SNE plot in **b**)

### Intrahepatic tumor ECs formed a distinct subpopulation, and adjacent normal ECs were affected by the presence of tumor

To induce liver cancer in situ in immune-competent mice, we employed a hydrodynamic delivery (HDD) approach targeting oncogenic pathways in hepatocytes. Activated Kras (mKrasG12D) and deletion of p53 (CRISPR-sgTrp53) were delivered to immune-competent C57BL/6 and BALB/c mice as well as immune-compromised SCID mice, via HDD of plasmid DNA. Mice subjected to HDD developed multiple tumors throughout the liver. Histological examination of these tumors revealed a mixed hepatocellular/cholangiocarcinoma phenotype (Fig. 2a), similar to what has been reported in some liver cancer patients [11]. Certain tumor regions showed a solid trabecular structure, as typically observed in human hepatocellular carcinoma, whereas other regions displayed bile duct differentiation features, as evidenced by the positive staining for cytokeratin 19, and featured a Masson-positive stromal reaction, similar to human cholangiocarcinoma. Tumor-bearing livers were either used undissected (resulting in a mix of cells from tumor and adjacent normal tissue) or dissected macroscopically into tumor and adjacent normal tissue. It is noteworthy that the dissected adjacent normal portion of a liver might have contained small tumors that were not visible by gross inspection. Similarly, dissected tumor tissue might have contained small portions of adjacent normal tissue.

When comparing ECs from HDD-induced liver tumors to those from normal liver, we initially focused on one strain of immune-competent mice. A total of 356 single ECs from tumors or adjacent normal tissues of C57BL/6 mice were identified and combined with the previously collected 471 ECs from naïve normal liver for cell-clustering analyses (Fig. 2b). As previously, ECs from CV and PV formed distinct clusters (cluster 3 and 4), but cells segregated by sample type within the CV cluster. Sinusoidal ECs were separated into two clusters: one cluster (cluster 0) predominantly composed of cells from naïve normal liver; the other (cluster 1) mainly composed of cells from tumor-adjacent normal liver. ECs from liver tumors formed a distinct cluster (cluster 2). Examination of cluster-specific marker genes showed that tumor ECs expressed a unique set of genes such as *Col18a1* and *Aplnr*, but also shared a number of expressed genes with venous ECs, especially with PV, such as *Cd63, Ehd4*, and *Cd200* (Fig. 2c and d, Supplementary Fig. 3a, and Supplementary Table 2). In contrast, tumor EC transcriptomes showed minimal overlap with sinusoidal EC transcriptomes. As previously reported, significantly higher numbers of genes were detected in tumor ECs than normal ECs (Fig. 2e) [12]. Genes preferentially expressed by tumor ECs were enriched in functions including angiogenesis, cell proliferation, cell-cell adhesion, and response to cytokines (Fig. 2f).

To determine whether the presence of an adaptive immune system affected the pattern of tumor EC gene expression, HDD-induced liver tumors from immune-deficient SCID mice were also profiled. As seen in immune-competent mice, a distinct cluster of tumor ECs was observed in HDD-induced tumors in SCID mice, along with similar tumor EC-specific genes and their associated functions (Supplementary Fig. 3b–d).

Many gene expression differences were observed between naïve liver and so-called adjacent normal liver tissue, suggesting that the tumor influenced the transcriptome of adjacent normal stromal cells including ECs. For example, *Selp* was increased in SECs and induced in PV from tumor-bearing livers. This result was supported by RNAScope findings (Figs. 1f and 2d). Another molecule *Lrg1*, a mitogen demonstrated to promote angiogenesis in the presence of TGF-β1 [13], was highly expressed in tumor ECs and upregulated in adjacent normal ECs (Fig. 2g). The presence of tumor also induced upregulation of *Sema3d*, which encodes the ligand for plexin D1 and is involved in angiogenesis, in adjacent normal ECs (Fig. 2g). We also observed that the transcriptome alterations in tumor-adjacent normal vs. naïve normal liver differed between C57BL/6 and SCID mice (Supplementary Table 2), although there was overlap in certain differential genes. For example, upregulation of *Serpina3h* was only observed in the liver SECs of C57BL/6 mice (Fig. 2c). With the same cutoffs applied, a lower number of differential genes between adjacent normal and naïve normal liver were detected in CV ECs compared to SECs (Supplementary Table 2). However, there were fewer CV and PV cells than SECs, which limited the power of this type of analysis. In summary, the presence of a liver tumor led to transcriptome changes in ECs of the adjacent normal liver region.

### Minimal impact of host immune status on tumor EC transcriptome

**Fig. 3:**
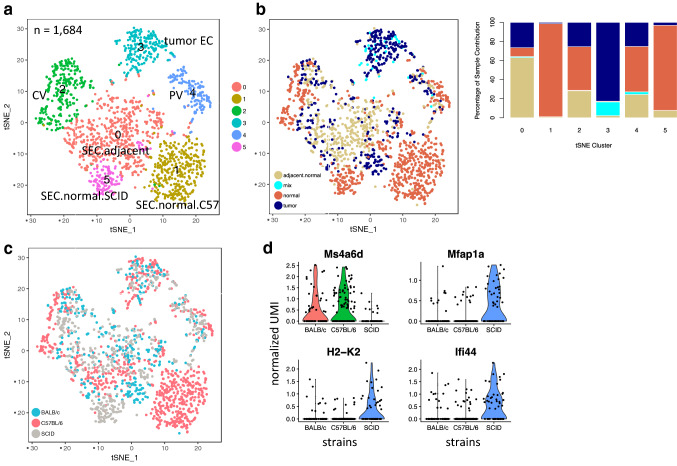
Tumor ECs derived from HDD-induced liver tumors of various genetic backgrounds were intermixed. **a** t-SNE plot of combined endothelial single cells collected from tumor-bearing and naïve liver tissues from C57BL/6, BALB/c and SCID mice. Cells are colored by identified clusters which were annotated by markers, sample type and strain information. **b** Left, t-SNE plot shown in 3a colored by sample type; Right, percentage of cell contribution from different sample types. **c** t-SNE plot shown in 3a colored by mouse strain. Liver tumor ECs grown in different mouse strains form one distinct cluster and were well-intermixed within the cluster. **d** Violin plot showing expression of select differentially expressed genes in tumor ECs from immune-competent and immune-compromised mice

Since normal ECs from C57BL/6 and SCID mice showed substantial transcriptional differences, we combined ECs derived from both tumor-bearing and normal (naïve and adjacent normal) livers from C57BL/6, BALB/c and SCID mice and re-performed cell clustering (Fig. 3). Over 1600 ECs clustered primarily based on EC subtypes, such as cluster 2 and 4 representing CV and PV ECs, respectively. SECs roughly formed three clusters representing sinusoids from naïve normal C57BL/6, naïve normal SCID, and adjacent normal liver tissues from all three mouse strains (Fig. 3a–c). Again, tumor ECs collectively formed a distinct cluster (Fig. 3b). Within the tumor EC cluster, cells from different strain backgrounds mixed well and did not display disparity (Fig. 3c). Nevertheless, we queried for differentially expressed genes in tumor ECs between immune-competent (C57BL/6 or BALB/c) and immune-compromised (SCID) strains and found only a few genes with significant scores (adjusted *P* value < 0.01 and fold change > 2) (Fig. 3d and Supplementary Table 2). Additionally, fewer genes overlapped between the two comparison results. Thus, it is reasonable to conclude that intrahepatic tumor ECs were minimally impacted by immune status of the host tissue.

### Minimal impact of intrahepatic tumor type on tumor EC transcriptome

**Fig. 4:**
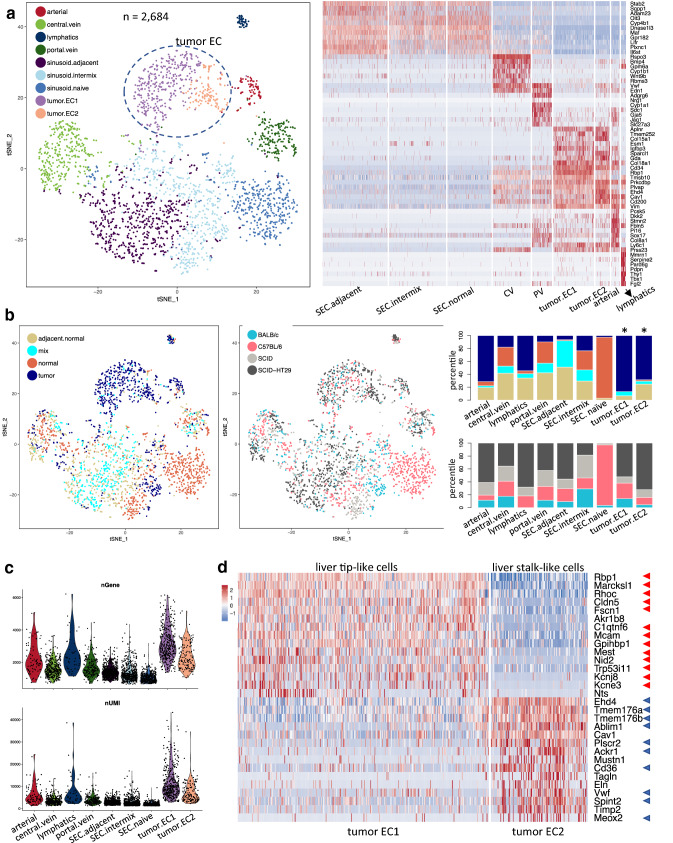
Identification of arterial, lymphatic, tumor tip and stalk subpopulations by combined analysis of ECs in naïve and tumor-bearing liver tissues. **a** Left, t-SNE plot of ECs derived from naïve normal liver, HDD-induced tumor-bearing and HT-29-bearing liver tissues. Nine subpopulations were identified and annotated as arterial, CV, lymphatics, PV, 3 sinusoids subpopulations, and two tumor EC subpopulations (tumor.EC1 and tumor EC.2). Right, heatmap of top 8 subpopulation-specific genes. **b**. Left, t-SNE plot (corresponding to Fig. 4a) colored by sample type or mouse strain; Right, stacked bar plot of percentage of contribution by sample type or mouse strain. **c**. Gene and UMI counts across the nine subpopulations (**a**). **d**. Heatmap of top 15 genes enriched in liver tumor tip-like (*n* = 373) and stalk-like (*n* = 133) EC subpopulations. Red and blue arrows indicate genes also identified as tip-like and stalk-like cell markers in s.c. tumors, respectively

To determine whether the tumor type affects tumor EC phenotype, we utilized a second intrahepatic tumor model, where human HT-29 colorectal cancer cells, which we used previously for s.c. tumor studies [1], were implanted directly into the liver parenchyma. Such a model has been used to represent metastatic colorectal cancer [14]. Intrahepatic HT-29 tumors grew as a single mass in the liver and displayed features of a well differentiated CRC, as evidenced by neoplastic cells with a glandular differentiation and mucinous material in the glandular lumens, as well as a prominent stromal compartment (Supplementary Fig. 4a). To increase the power of the analysis, ECs from all sample collections including naïve normal liver, adjacent normal liver, and intrahepatic HDD-derived and HT-29 tumors were merged and subsequently clustered into nine subpopulations (Fig. 4a). At first glance, two clusters stood out because they were almost exclusively composed of cells from tumors (Fig. 4b). Therefore, these clusters were designated as tumor ECs. While CV- and PV-derived ECs showed one distinct cluster each, the SEC subpopulation was further divided into three clusters despite a shared common gene signature: one cluster was almost exclusively comprised of cells from naïve livers (only collected from C57BL/6 and SCID mice), which was named sinusoid.naïve, one cluster mainly contained ECs from tumor-bearing livers (adjacent normal or mix) and thus named sinusoid.adjacent, and one cluster designated as sinusoid.intermix contained cells from both naïve and tumor-bearing livers (Fig. 4a). Additionally, two new distinct clusters emerged. They were annotated as arterial ECs and lymphatics based on known marker genes (Fig. 4a left). For example, *Stmn2* and *Sox17* were expressed in arterial cells [1] and *Mmrn1* and *Pdpn* are lymphatic-specific [1]. We further validated lymphatic-specific expression of *Tbx1* by RNAScope (Supplementary Fig. 4b). Lymphatic ECs, which were mostly tumor-associated (Fig. 4b), showed a higher gene count per cell (Fig. 4c) than ECs from normal liver.

The cumulative 506 tumor ECs derived from intrahepatic tumors (HDD-induced and HT-29 tumors) separated into two distinct subpopulations (Fig. 4a). The top subpopulation-specific genes in clusters tumor.EC1 and tumor.EC2 highly overlapped with those previously identified as “tip-like” and “stalk-like” EC marker genes in s.c. tumors, respectively (Fig. 4d) [1]. For example, *Dll4* and *Notch4* expression was limited to tip-like cells, *Tgfbr3* was expressed in stalk-like cells, while both EC populations expressed similar levels of *Kdr* (*Vegfr2*) (Supplementary Fig. 4c).

Within the two tumor EC clusters, cells from various mouse strains and tumor types (mouse and human tumors) were well-intermixed in the t-SNE plot (Fig. 4b). Focusing on only SCID mice, we further checked gene expression differences in tumor ECs between HDD-derived and transplanted HT-29 intrahepatic tumors. Only a few genes showed preferential expression in one or the other intrahepatic tumor model (Supplemental Fig. 4d). Thus, the tumor type exerted minimal influence on the tumor EC transcriptome profiles in liver.

#### Integrated analysis of normal and tumor ECs from different organs

**Fig. 5:**
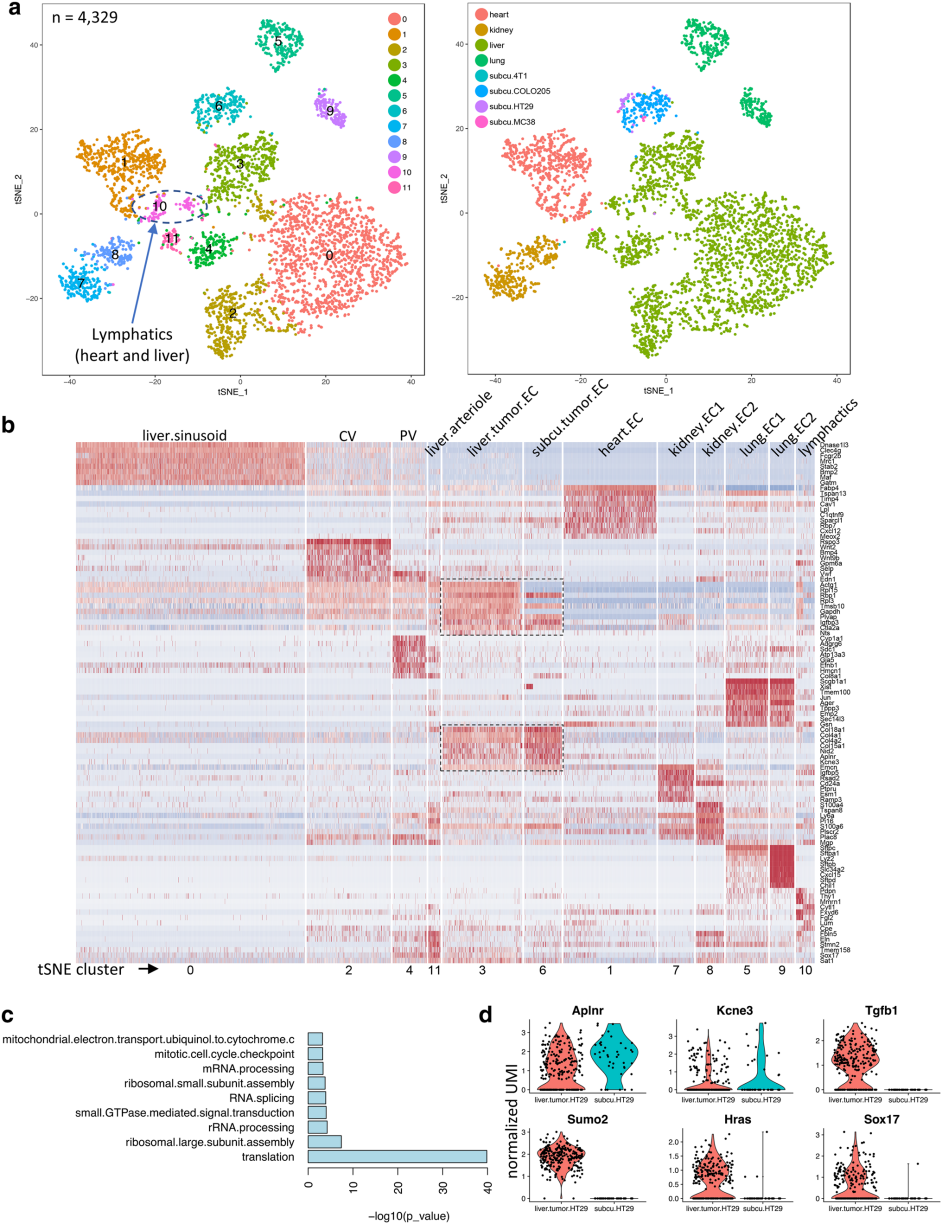
Comparison of normal and tumor ECs from different host organs revealed tissue-specific signatures in ECs. **a** t-SNE plot of a collection of ECs from different normal tissues (liver in this study *n* = 719, heart *n* = 629, lung *n* = 401 and kidney *n* = 386) as well as tumors (intrahepatic tumors in this study and s.c. tumors *n* = 240). ECs are colored by clusters (left) or by tissue of origin and tumor types (right). **b** Heatmap showing top 10 cluster-enriched genes in different subpopulations (as in 5a). Gene signatures distinctive to tumor ECs are highlighted by dashed boxes. **c** Functions enriched in genes preferentially expressed by intrahepatic HT-29 tumor ECs compared to s.c. HT-29 tumor ECs. **d** Violin plots showing conserved tumor EC genes (*Aplnr* and *Kcne3*) with similar expression levels in intrahepatic and s.c. HT29 tumors and genes exclusively expressed in intrahepatic HT-29 tumor ECs (*Tgfb1*, *Sumo2*, *Hras*, *Sox17*)

The clear separation of tumor ECs from naïve/adjacent normal ECs, and the observed conservation of tip- and stalk-like gene signatures in tumor ECs from s.c. and liver tumors, prompted us to look for commonalities that could serve as tumor EC-specific markers. Consequently, we performed an integrated analysis by combining our liver and heart [1] data with published single cell data on ECs from normal mouse tissues including lung [15] and kidney [16] obtained using comparable single-cell sequencing techniques. Even when sequence read coverages in ECs from different sources were comparable, the number of genes detected could vary significantly (Supplementary Fig. 5a). ECs from different normal organs (heart, lung, kidney, liver) clustered based on tissue of origin (Fig. 5a). Most normal organs further displayed EC subpopulations, such as liver (cluster 0, 2, 4, 11), kidney (cluster 7, 8) and lung (cluster 5, 9), whereas heart ECs formed one cluster (cluster 1) at the applied clustering resolution. At the applied cluster resolution, lymphatic ECs from heart and liver mapped into one cluster (cluster 10), indicating substantial similarity, which was further supported by a high-correlation coefficient score in transcriptome (Supplemental Fig. 5b). Tumor ECs clustered away from normal ECs. Despite conservation of tip- and stalk-like genes, s.c. tumor ECs formed a separate cluster (cluster 3) from intrahepatic tumor ECs (cluster 6).

Because the ECs in the combined analysis above were pooled from different sources, batch effects should not be ignored. However, because ECs in the s.c. tumor cluster (cluster 3) were collected from different xenograft models and were profiled in separate 10× Genomics single cell runs, the most influential factor on EC transcriptome appears to be the host tissue. Each cluster displayed distinct tissue-specific and subpopulation-specific gene signatures (Fig. 5b), which were deprived of the usual batch-related genes such as *Jund*, *Klf* and ribosomal genes. Although common tumor EC genes such as *Nid2* and *Col15a1*, were detected in s.c. and liver tumor-derived ECs, two distinct clusters formed based on the site of tumor growth (i.e., s.c. and liver). Collectively, ECs express common endothelial lineage markers but also carry tissue- and cell state-specific imprints (proliferation, metabolism, etc.).

Following up on this observation, we focused on HT-29 tumors in SCID mice and assessed differentially expressed genes in ECs derived from intrahepatically and subcutaneously grown tumors. Many genes were preferentially expressed in intrahepatic tumor ECs, whereas few genes were uniquely expressed in s.c. tumor ECs (Fig. 5b). Although the majority of the liver-specific genes appeared to be involved in house-keeping functions (Fig. 5c and Supplementary Table 3), there were a number of genes such as *Tgfb1*, *Hras*, *Sumo2* and *Sox17* that are involved in cellular functions beyond housekeeping (Fig. 5d). These results showed that the host organ, but not the tumor type, exerted significant influence on EC phenotypes in liver tumors. Taken together, tumor ECs carry conserved as well as host organ-imposed gene signatures.

#### Chimeric myeloid-endothelial cell type present in tumor-bearing livers

**Fig. 6:**
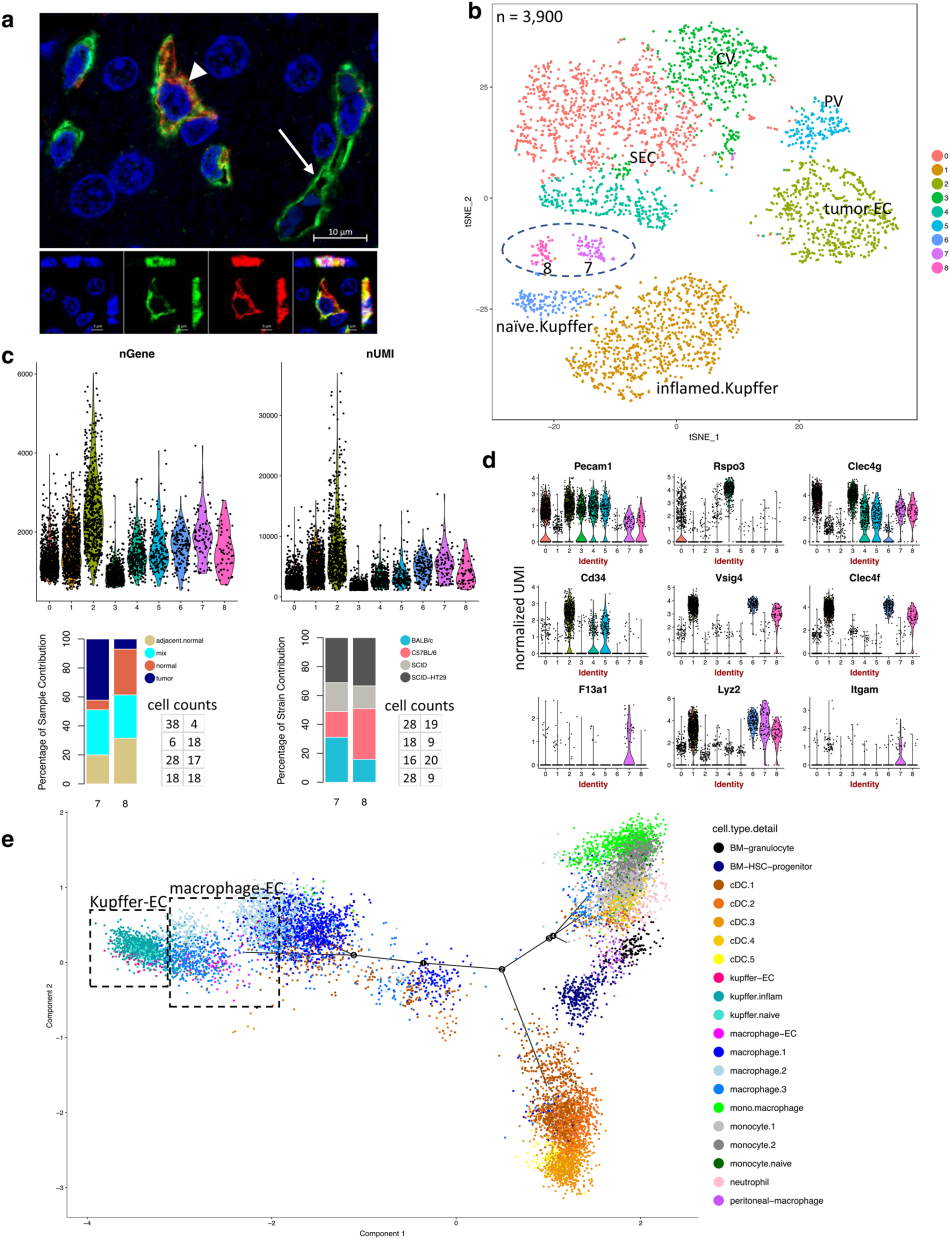
Identification and characterization of chimeric ECs in normal and tumor-bearing liver tissues. **a** Representative IHC image of the pan-EC marker CD31 (green), the Kupffer cell marker Clec4f (red) and the nuclear marker DAPI (blue). Top: Compressed z-stack (deconvoluted) of confocal image, arrow head indicates double-positive cell, arrow indicates single-positive vascular structure; Bottom: single optical plane with orthogonal view of single channels and overlay of double-positive cell. **b** Combined clustering analysis on Kupffer cells and ECs revealed two distinct clusters (7 and 8) between ECs and Kupffer cells. **c** Top, gene and UMI counts per cluster (**b**); Bottom, percent contribution of cells to cluster 7 and 8 by sample type and mouse strain, respectively, with exact cell counts next to the plot in the same order as in the stacked bar plot. **d** Violin plots showing that cluster 7 and 8 express Kupffer/macrophage and EC marker genes as well as myeloid genes at various levels. The numbers on x-axis match to the cluster numbers in the t-SNE plot (**b**). **e** Trajectory analysis of all myeloid, Kupffer-EC and macrophage-EC subpopulations. Kupffer-EC (red) and macrophage-EC (pink) cells are highlighted in dashed boxes

When assessing EC marker gene expression in all single cells collected from the liver, we noticed that a subpopulation of the Kupffer cell cluster also expressed conventional EC marker genes (Supplemental Fig. 6a–c). Based on gene and UMI counts, these cells did not appear to be doublets (Supplemental Fig. 6d). In support of the existence of chimeric cells, double-positive cells expressing the EC marker CD31 (Pecam1) and the Kupffer cell marker Clec4f were detected by IHC (Fig. 6a). These cells were often detected close to blood vessels without integrating into the vessel. To further investigate these cells, we combined all Kupffer cells and ECs for re-clustering. Two subpopulations (cluster 7 and 8) emerged between ECs (cluster 0, 2, 3, 4 and 5) and Kupffer cells (cluster 1 and 6), which displayed mixed molecular phenotypes (Fig. 6b). In particular, cells in cluster 7 expressed a higher number of genes, which is often seen in tumor ECs, and were more prevalent in tumor-bearing livers than naïve livers (*P* value < 0.001 by Fisher Exact test) (Fig. 6c). The host mouse strain did not affect the presence of these two populations (Fig. 6c). All cells from clusters 7 and 8 expressed EC marker genes, such as *Pecam1, Clec4g, Rspo3, Egfl7, and Robo4*, as well as monocyte/macrophage markers, such as *Csf1r* and *C1qa* (Fig. 6d and Supplementary Fig. 7a).

Furthermore, there were differences between cluster 7 and 8. In particular, cells in cluster 7 showed preferential expression of monocyte/macrophage markers *F13a1* and *Itgam (Cd11b)*, whereas cells in cluster 8 expressed higher levels of Kupffer cell markers *Clec4f* and *Vsig4* (Fig. 6d and Supplementary Fig. 7b, c). Further trajectory and cluster tree analyses suggested that clusters 7 and 8 possessed more EC transcriptome properties than Kupffer cell properties (Supplementary Fig. 7d). Altogether, this led us to name cluster 7 “macrophage-EC” and cluster 8 “Kupffer-EC”. To further investigate the myeloid signal in these chimeric ECs, we first classified all myeloid cell populations and subpopulations in our dataset (Supplementary Fig. 8 and Supplementary Table 4). Next, we constructed a trajectory including all myeloid cells (except plasmacytoid dendritic cells, i.e., pDC) and the chimeric ECs, which revealed that the “Kupffer-ECs” (red) were most closely related to naïve and inflamed Kupffer cells (teal and turquoise), whereas the “macrophage-ECs” (pink) clustered with macrophages (different shades of blue), all of which were located at the same end of the trajectory. A closer look at the EC features of “Kupffer-ECs” and “macrophage-ECs” revealed that specific CV and PV marker genes described above were barely expressed in either subpopulation (Fig. 6d; *Rspo3* as an example of a CV marker). This observation suggests that chimeric ECs represent an intermediate phenotype between Kupffer cells or macrophages and sinusoidal ECs.

## Discussion

In this study, we performed transcriptome profiling on single cells from normal and tumor-bearing livers and studied endothelial cell heterogeneity at the single-cell level (summary of sample collection in Supplemental Table 5). We monitored batch effect by performing multiple sequencing runs on the same sample type and concluded that batch effect is of minimal concern and will not affect our conclusions. Analysis of ECs from normal livers revealed zonation-related gene expression patterns along the PV-CV liver zonation axis. We also observed that mouse strain affected EC gene expression signatures. Our data showed that genes highly expressed in SECs of C57BL/6 mice were significantly associated with immune-related pathways, such as genes involved in MHC class II antigen processing and presentation including *Cd74*, *H2-Ab1*, *Ctsb*, and *Lgmn*, which has been related to induction of immune tolerance [17]. However, whether the strain-specific genes detected in ECs between C57BL/6 and SCID were associated with the immune status of the mice needs further experimental validation. Despite the fact that a more systematic approach is needed to identify and assign relevance to EC gene signatures that are influenced by immune status, our data did provide some insights into the influence of the mouse immune status on myeloid cell populations. For example, subpopulations within macrophages were apparently driven by strain differences in a tumor setting. Similarly, it has been reported that the immunologic phenotypes of commonly used inbred mouse strains, such as BALB/c and C57BL/6, are quite divergent when used for tumor studies [18]. This raises the question, to what extent these differences affect non-immune stromal cells in the TME, such as ECs or fibroblasts.

Tissue samples derived from the non-tumor-bearing part of an organ are often used as control material when studying changes prevalent in the tumor tissue. However, a comprehensive analysis of bulk RNA from tumor, adjacent normal, and naïve (non-tumor-bearing organ) tissue data from the GTEx and TCGA datasets revealed that so-called adjacent normal tissue presents an intermediate state between naïve/healthy and tumor [6]. Similarly, at the single cell level, ECs from adjacent normal tissue in tumor-bearing livers had pronounced alterations in transcriptome compared to ECs from naïve livers. These data highlight that stromal cells in so-called adjacent normal tissue might already harbor tumor-induced changes.

Additional changes in EC phenotypes occurred in the actual tumor mass. The arterial and lymphatic EC populations appeared to expand, since these clusters were mainly composed of tumor-derived ECs. These observations were in agreement with previous reports that HCC or intrahepatic CRC tumors have increased lymphangiogenesis [19, 20], and that a switch to prominent arterial blood supply occurred at the stage of early HCC [21–23]. Interestingly, lymphatic ECs expressed a higher number of genes than blood vessel EC. In particular, cytokine and chemokine receptors were highly upregulated in lymphatic ECs, suggesting a close functional relationship between lymphatic ECs and immune cells. Tumor ECs form distinct clusters of tip-like and stalk-like endothelial cells, which share signatures with tip-like and stalk-like endothelial cells previously defined in s.c. tumors, indicating conserved angiogenic mechanisms during tumor angiogenesis. Notably, the influence of tumor type (induced vs. implanted HT-29) on tumor ECs was rather limited. Globally, liver tumor ECs did not express typical EC markers for SEC (*Clec4g*) and lost classical CV (*Rspo3*) and PV (*Adgrg6*) markers. However, tumor ECs carried a gene signature related to PV ECs (Fig. 2b and c).

Comparison of our single cell transcriptome profiling data with previously published EC signatures from normal liver and liver tumors revealed significant overlap. For example, Seaman et al. [24] identified normal and tumor EC-specific signatures in mouse liver tumor models using a SAGE platform on pooled ECs. Halpern et al. [8] used a paired-cell sequencing strategy to infer the expression zonation of liver ECs. The overlapping gene expression signature between these studies support our EC cluster annotation. However, the analysis of individual cells in the current study provided additional granularity and depth of information not only on ECs but also acrosss various other cell types and subtypes in tumors.

We identified a set of chimeric myeloid-endothelial cells in our study. Upon further resolution, these chimeric cells could be classified as either Kupffer-ECs (expressing both Kupffer and EC markers) or macrophage-ECs (expressing macrophage and EC markers). In our studies, these cells were detected at a rate of about five percent (147 out of 2,684 ECs). Based on strain and sample type distribution compared to other EC cell types, we speculate that the Kupffer-EC population pre-exists in naïve liver tissue, whereas the macrophage-EC population is less common in naïve liver, but expands when a tumor is present, since these cells were significantly enriched in tumor-associated samples and were phenotypically closer to tumor-associated macrophages.

Contributions of bone marrow (BM)-derived progenitor cells to the formation of liver sinusoidal vessels have been reported in liver transplantation experiments under varied conditions [25–30]. For example, BM-derived mononuclear cells were reported to contribute to liver sinusoidal vessels after liver EC injury by irradiation, but not during liver regeneration after partial hepatectomy in mouse models [28]. In other studies, myeloid progenitor cells differentiated into sinusoidal EC regardless of liver injury [25]. During liver development in embryogenesis, a dual mechanism of vascular expansion involving proliferation of existing endothelial cells as well as incorporation of erythro-myeloid precursors has been described [27]. In tumor settings, the contribution of BM-derived endothelial progenitor cells to tumor angiogenesis is less clear [31]: on one hand BM-derived endothelial progenitor cells were reported to contribute to tumor angiogenesis in both direct and indirect ways [32], whereas other studies showed that the recruited BM-derived cells did not play a role in tumor angiogenesis [33, 34]. Results from our single cell analyses indicate the macrophage-EC population, but not the Kupffer-EC population, is significantly enriched in tumor-bearing livers, possibly suggesting a role for this cell population in tumor angiogenesis or other aspects of the tumor microenvironment.

Previous studies were not able to fully characterize the expression profile of these cells, but our application of single cell approaches now provides such signatures in an unbiased way. One of the EC markers expressed in both Kupffer-ECs and macrophage-ECs is *Tek/Tie2* (Supplementary Fig. 9), which leads us to speculate that these cells represent a rare cell population in tumor-bearing mice known as pro-angiogenic, pro-metastatic Tie2-expressing monocytes [35–41] also found in HCC-bearing liver tissue [42, 43]. Pucci et al. reported that the expression profile of Tie2-expressing monocytes clearly indicated a monocyte/macrophage lineage rather than an EC lineage [40]. In our study, the chimeric cells initially clustered with Kupffer cells when all cells in the liver were analyzed, but when directly compared with only Kupffer cells and ECs (including all EC subpopulations in normal and tumor-bearing livers) using trajectory and cluster tree analyses, the chimeric cells displayed more similarity to ECs. Further, our data suggest that distinct subpopulations of Tie2-expressing myeloid cells, such as Tie2-positive Kupffer-ECs and macrophage-ECs in the liver, exist. This raises the question whether Tie2-expressing cell subpopulations could differentially affect tumor angiogenesis and metastasis.

Taken together, our results highlight the commonality of tumor ECs across different sites of tumor growth (intrahepatic vs. subcutaneous) and across different origins of tumors within the liver (induced liver cancer vs. CRC metastases). In addition, our studies further illustrate a clear difference between naïve and adjacent normal ECs and delineate two chimeric myeloid-EC cell types that might play a role in tumor angiogenesis.

## Methods

### In vivo models

Animal studies were performed in accordance with Regeneron’s Institutional Animal Care and Use Committee guidelines. HT-29 tumor cells [American Type Culture Collection (ATCC)] were authenticated in 2012 using the STR Profile Testing by ATCC. Micro-ultrasound (Vevo2100, VisualSonics) was used for image-guided intrahepatic implantation of 10^6^ HT-29 cells in Matrigel and longitudinal tumor growth monitoring (largest tumor diameter). Tumor-bearing livers were harvested at 10–30 mm^2^. For HHD-induced liver tumors, 50 µg of plasmids encoding mKras (G12D), Cas9 and sgTrp53 or mKras (G12D), Cas9 and sgRNA (empty vector) were combined and diluted in 0.9% NaCl to a volume ~ 10% of the mouse body weight and injected into the tail vein over six to ten seconds [44]. Tumor-bearing livers were harvested when sizeable tumors were present. After harvest, tissue was processed for histological analysis and single-cell sequencing.

### RNAScope and immunohistochemistry

Previously described IHC procedures [45] were used on 4 µm tissue sections. Specifically, after citrate antigen retrieval, sections were exposed to 0.3% H_2_O_2_ in methanol, TNT blocking buffer (Perkin Elmer), primary antibodies (anti-CD31 Ab (ab28364, Abcam), anti-CLEC4F Ab (AF2784, R&D Systems)) and detection agents (CD31: polymer HRP anti-rabbit followed by opal 520 fluorophore amplification reagent (PerkinElmer); CLEC4F: Cy-3 donkey anti-goat (Jackson Immuno Research). Nuclear stain: DAPI. Previously described RNAScope method [1] was used on 4 µm tissue sections using probes from ACD Bio.

### Single cell preparation for sequencing

Naïve and tumor-bearing liver tissues were processed with a consistent protocol as described previously [1] with an enzymatic collagenase/DNase treatment time of 13 min and PharMLyse (BD Biosciences) treatment of 5 min (tumor) and 10 min (liver, mix). HT-29 tumor single cell suspensions underwent a tumor cell depletion step as previously described [1].

Our single cell data were derived from multiple 10× Genomics sequencing runs. To benchmark batch effect in our data, each sample type (i.e. cells from naïve liver, tumor, adjacent normal or a mix type under certain genetic background) was subjected to multiple 10× Genomics runs spread across different days. Samples representing various samples types were collected on the same day and subsequently processed for sequencing. For the same sample type, batch effect was barely observed in cell clustering result.

### Single-cell sequencing

Single cells were resuspended in PBS + 0.04% BSA. Cellular suspensions (~ 6000 cells) were loaded on a Chromium Single cell Instrument (10× Genomics) to generate single cell GEMs. Single cell RNA-Seq libraries were prepared using version 2 Chromium Single cell 3’ Library, Gel beads & Multiplex kit (10× Genomics). Sequencing was performed on Illumina NextSeq500 using the following read length: 59 bp Read1 for transcript read, 14 bp I7 Index for Cell Barcode read, 8 bp I5 Index for sample index read, and 10 bp Read2 for UMI read.

### Bioinformatics analysis

#### Alignment, barcode assignment and UMI counting

The Single Cell Software Cell Ranger Suite version 2 was used to perform sample de-multiplexing, barcode processing and single-cell gene UMI (unique molecular index) quantification (http://software.10xgenomics.com/single-cell/overview/welcome). For single cells derived from HT-29 tumors, reads were mapped to both mouse and human genomes. Mouse cells were separated from human cells (HT-29 tumor cells) by preponderance of reads mapped to the mouse genome.

#### Single-cell RNA-Seq data QC

Mouse single cells were filtered for downstream analysis by the following criteria: the number of genes expressed (with at least one UMI count) is within a range between 500 and 6500, and mitochondria content is less than twenty percent of the total UMI count. Overall, 27,542 cells passed the QC steps, with 7449 cells derived from BALB/c, 8295 derived from C57BL/6, 5213 derived from SCID-HDD, and 6585 derived from SCID-HT-29 strain, respectively.

#### Clustering of cells and identification of cluster-specific genes

Mouse single cells from all sequencing batches were combined for the downstream analysis. All clustering analyses were done with Seurat v2 [46] (https://github.com/satijalab/seurat/) package by using from 300 to 500 highly variable genes and were displayed in t-distributed stochastic neighbor embedding (t-SNE) plots. FindAllMarkers function in Seurat was performed to call cell type-specific genes and differentially expressed genes. Top-ranked genes were ordered by fold change under a threshold of adjusted *P *value < 0.05 (Bonferroni correction). BuildClusterTree function in Seurat 2 package was applied to generate cluster trees.

#### Pseudotime and trajectory maps

The new reconstruction algorithms in Monocle 2 (http://cole-trapnell-lab.github.io/monocle-release/) was applied to single cell trajectory analysis in which cells were placed along the pseudotime tree but colored by cell type annotation. Either combined top 50 cell cluster-specific genes or top 500 most variable genes were used in building the branches, both led to essentially the same branch structure. The parameter of max components tested was between 10 and 12.

#### Statistical analysis

Conservation between cell types were assessed by Pearson correlation of mean UMI on expressed genes in each cell subpopulations. Function enrichment analyses on differentially expressed genes were performed using the Database for Annotation, Visualization and Integrated Discovery (DAVID 6.8, http://david.abcc.ncifcrf.gov). Comparison of the distribution of categorical variables in different groups was performed with the Fisher exact test using two-tailed *P* values.

## Electronic supplementary material

Below is the link to the electronic supplementary material.

Supplementary material 1 (PDF 32981.2 kb)

Supplementary material 2 (XLSX 56.6 kb)

Supplementary material 3 (XLSX 128.1 kb)

Supplementary material 4 (XLSX 33.4 kb)

Supplementary material 5 (XLSX 535.1 kb)

## Data Availability

Software used this manuscript are publicly available, mentioned in the methods section. Data generated from this study have been deposited to NCBI GEO repository with accession number GEO150226.
