# Supplementary material for: Heterogeneity and chimerism of endothelial cells revealed by single-cell transcriptome in orthotopic liver tumors
Source: Angiogenesis. 2020 May 21;23(4):581–97. doi: 10.1007/s10456-020-09727-9 (PMC7525283; doi:10.1007/s10456-020-09727-9)
Supplement: Supplementary file 1 — Supplementary material 1 (PDF 32981.2 kb) [file 10456_2020_9727_MOESM1_ESM.pdf]

Supplemental Fig. 1

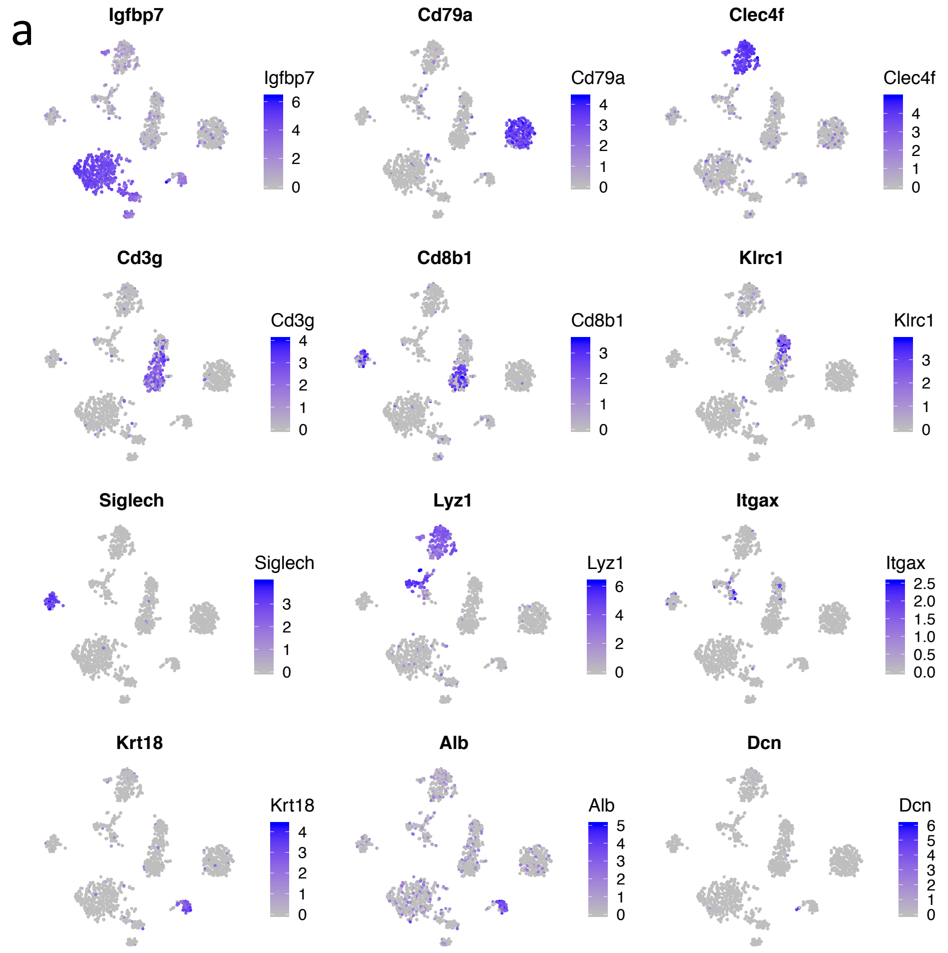

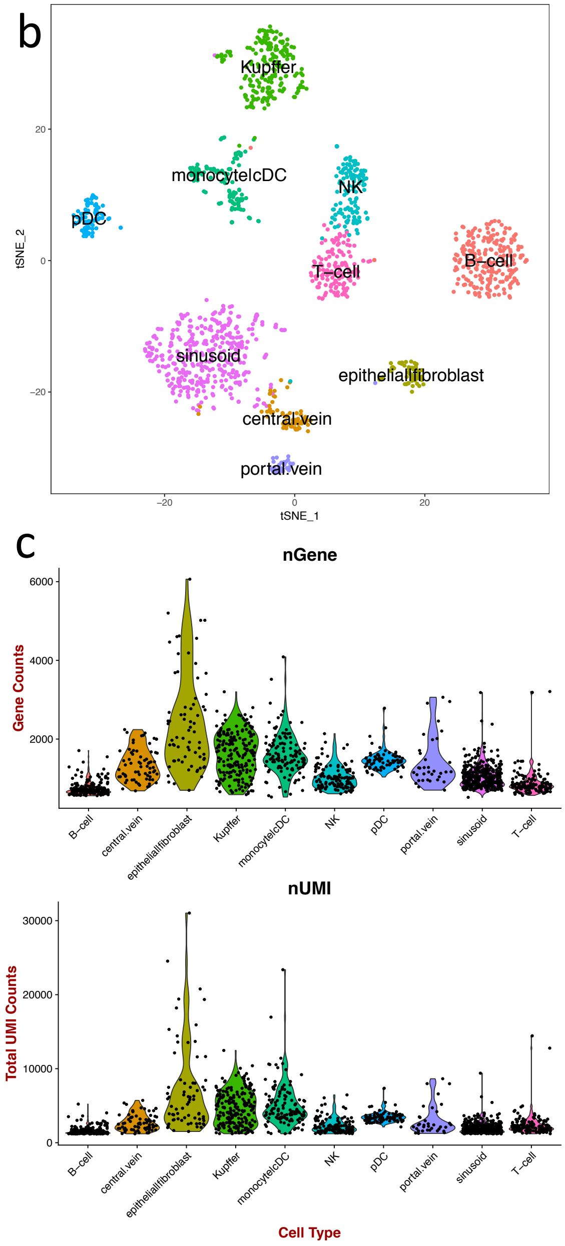

- Igfbp7 -- endothelial cell
- Cd79a -- B-cell
- Clec4f -- Kupffer cell

- Siglech -- pDC
- Lyz1 -- monocyte & macrophage
- Itgax -- (Cd11c), cDC

- Cd3g -- T-cell
- Cd8b1 -- Cd8+ T-cell
- Klrc1 -- NK cell

- Krt18 -- epithelial
- Alb -- hepatocyte
- Dcn -- fibroblast

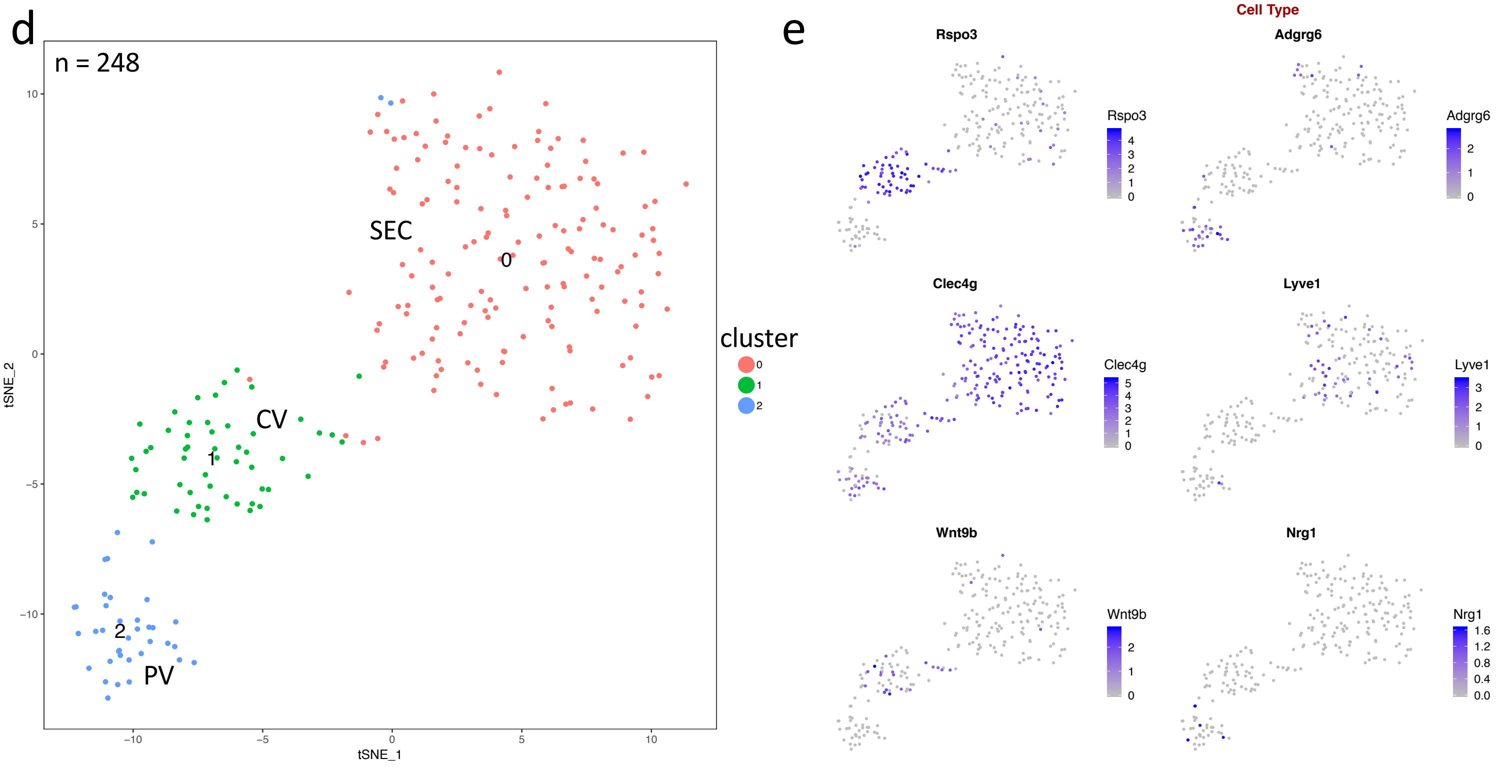

Supplemental Fig. 2

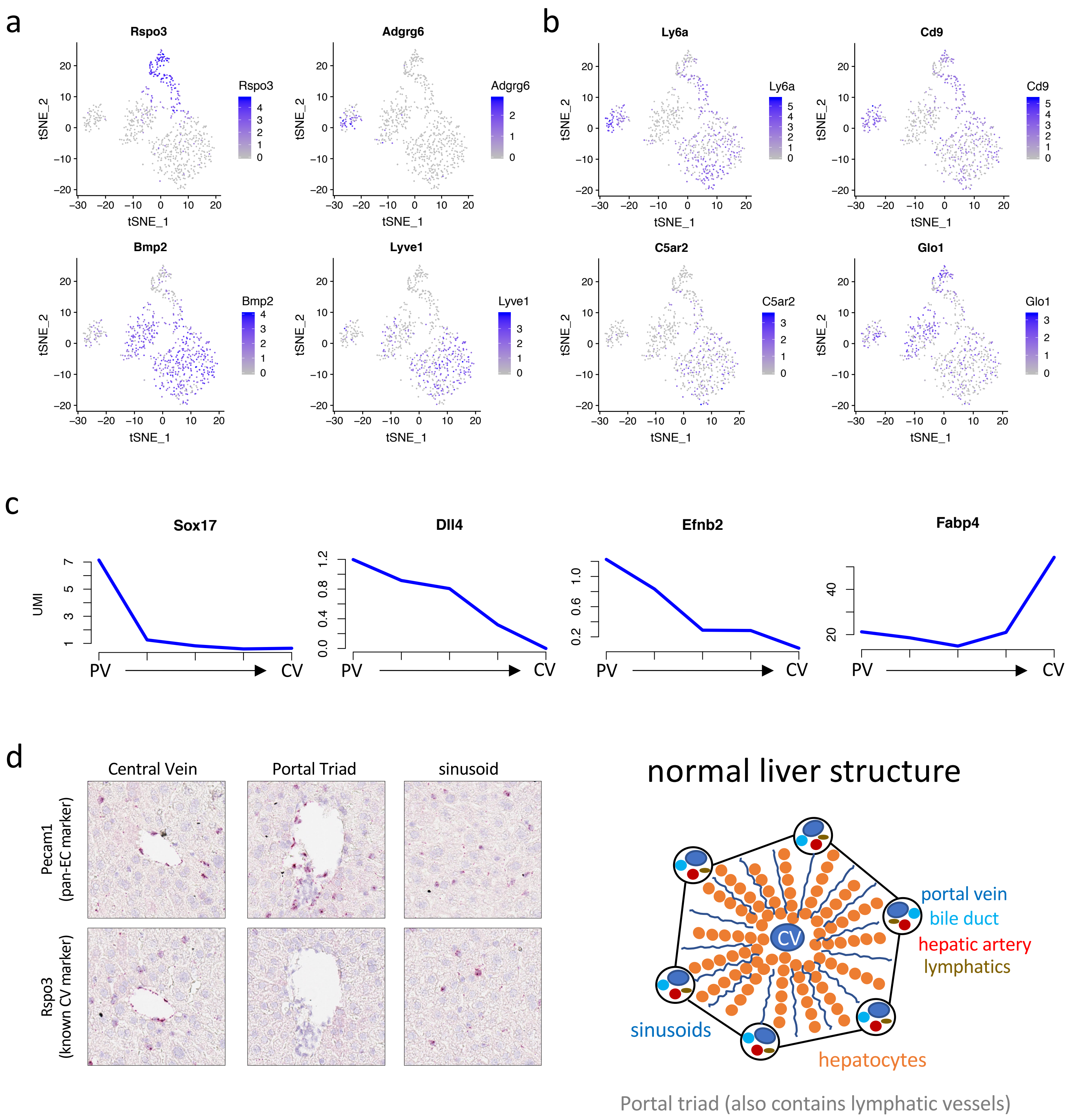

Supplemental Fig. 3

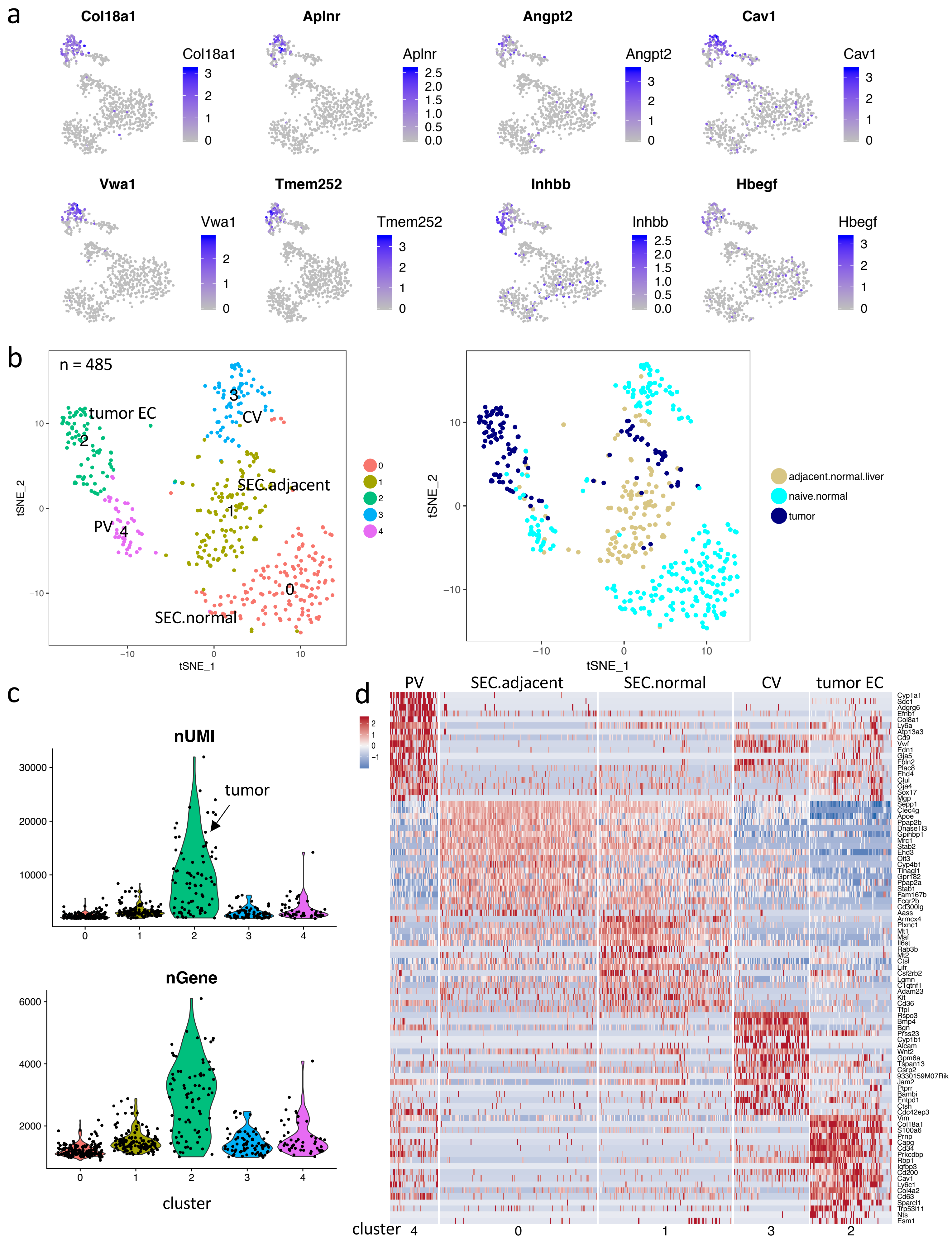

Supplemental Fig. 4

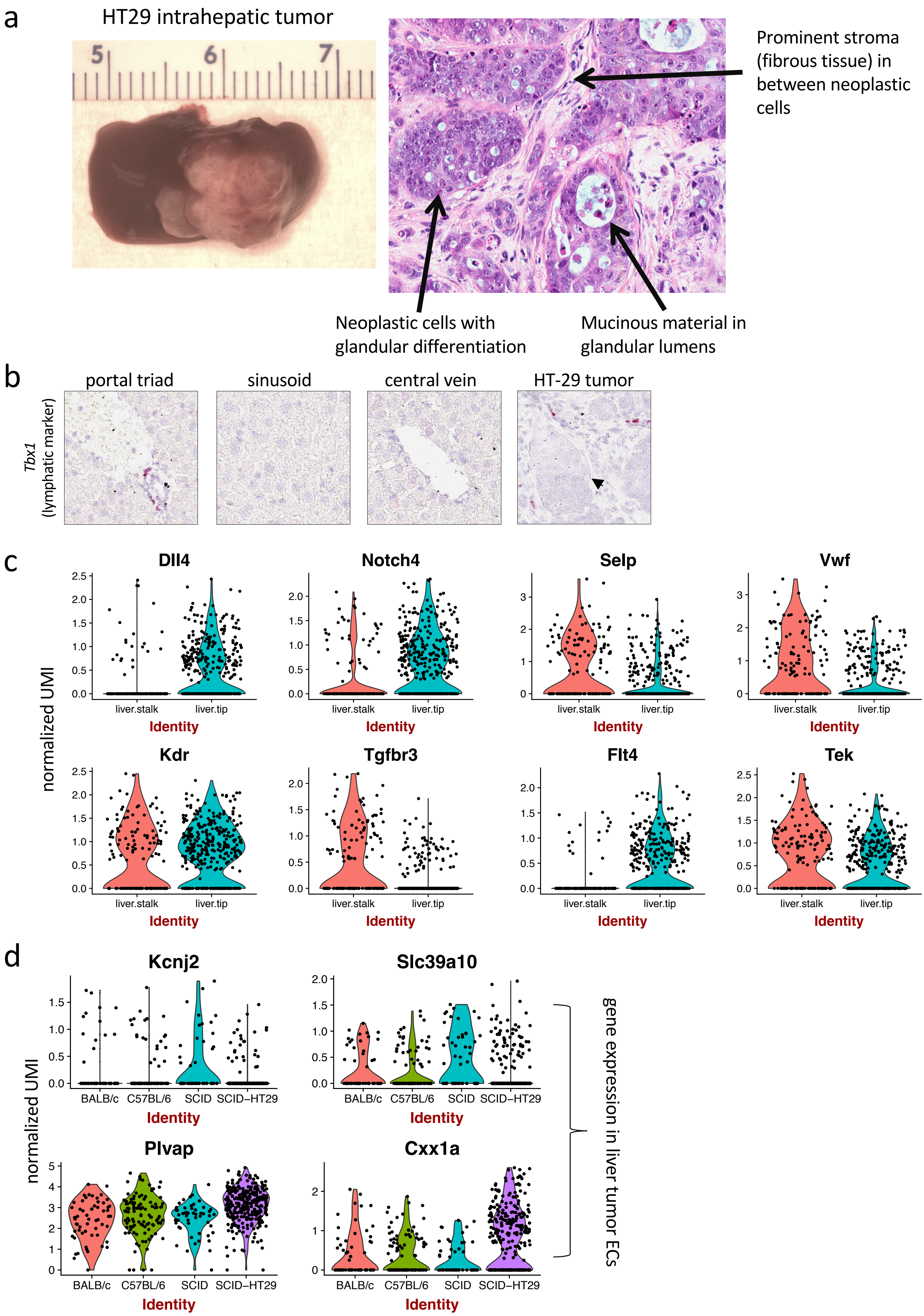

Supplemental Fig. 5

a

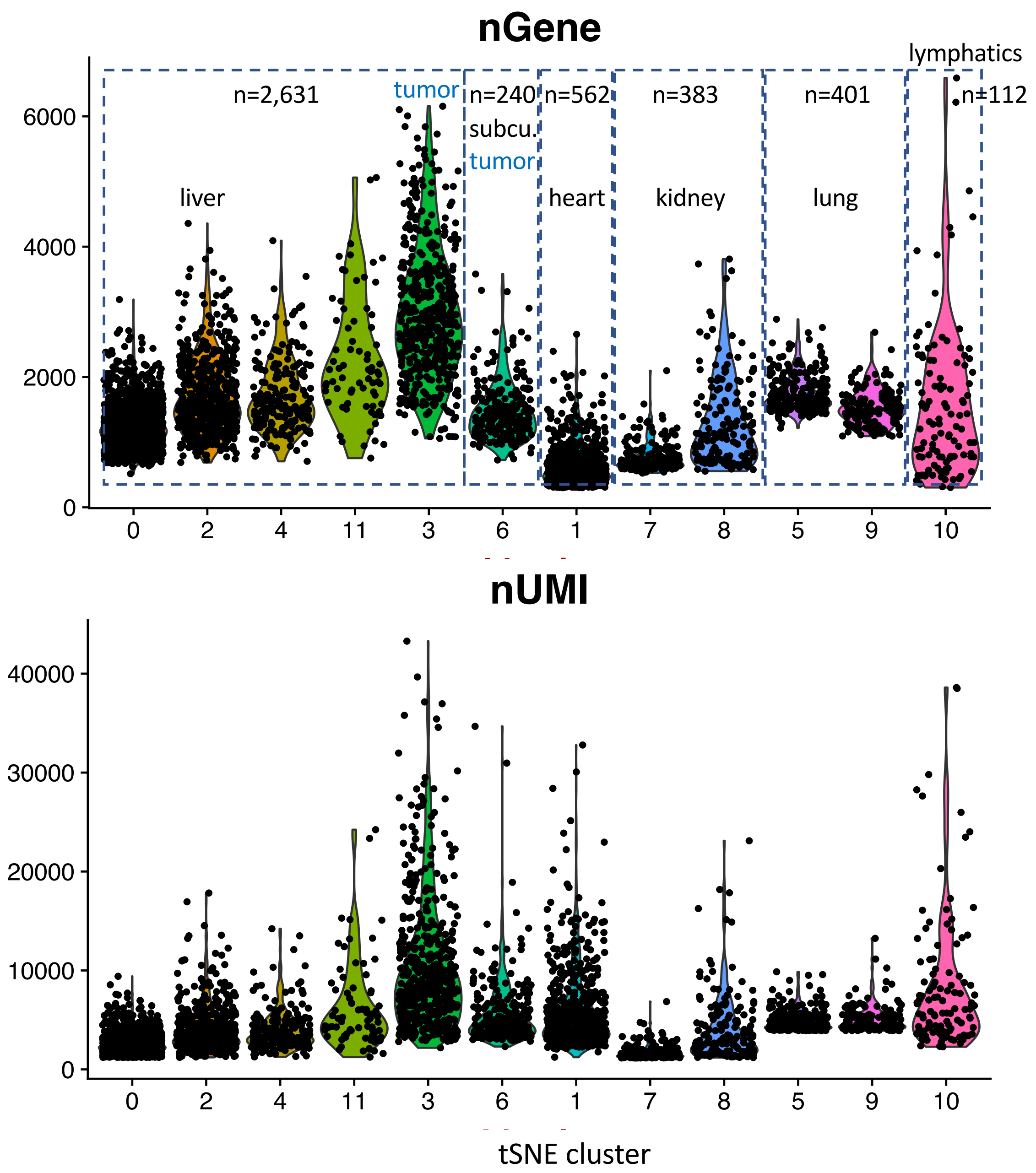

b

**liver vs. heart**	liver.sinusoid	liver.central.Vein	liver.portal.Vein	liver.arteriole*	liver.lymphatics*	liver.tumor.EC
heart.arteriole	0.4345	0.5144	0.6241	0.7182	0.3657	0.6574
heart.capillary	0.4572	0.5251	0.623	0.6775	0.3552	0.6743
heart.vein	0.4186	0.623	0.6453	0.6771	0.5439	0.5974
heart.lymphatics	0.3289	0.3968	0.4666	0.4846	0.8848	0.4668
subcu.tumor.EC						0.6633

Supplemental Fig. 6

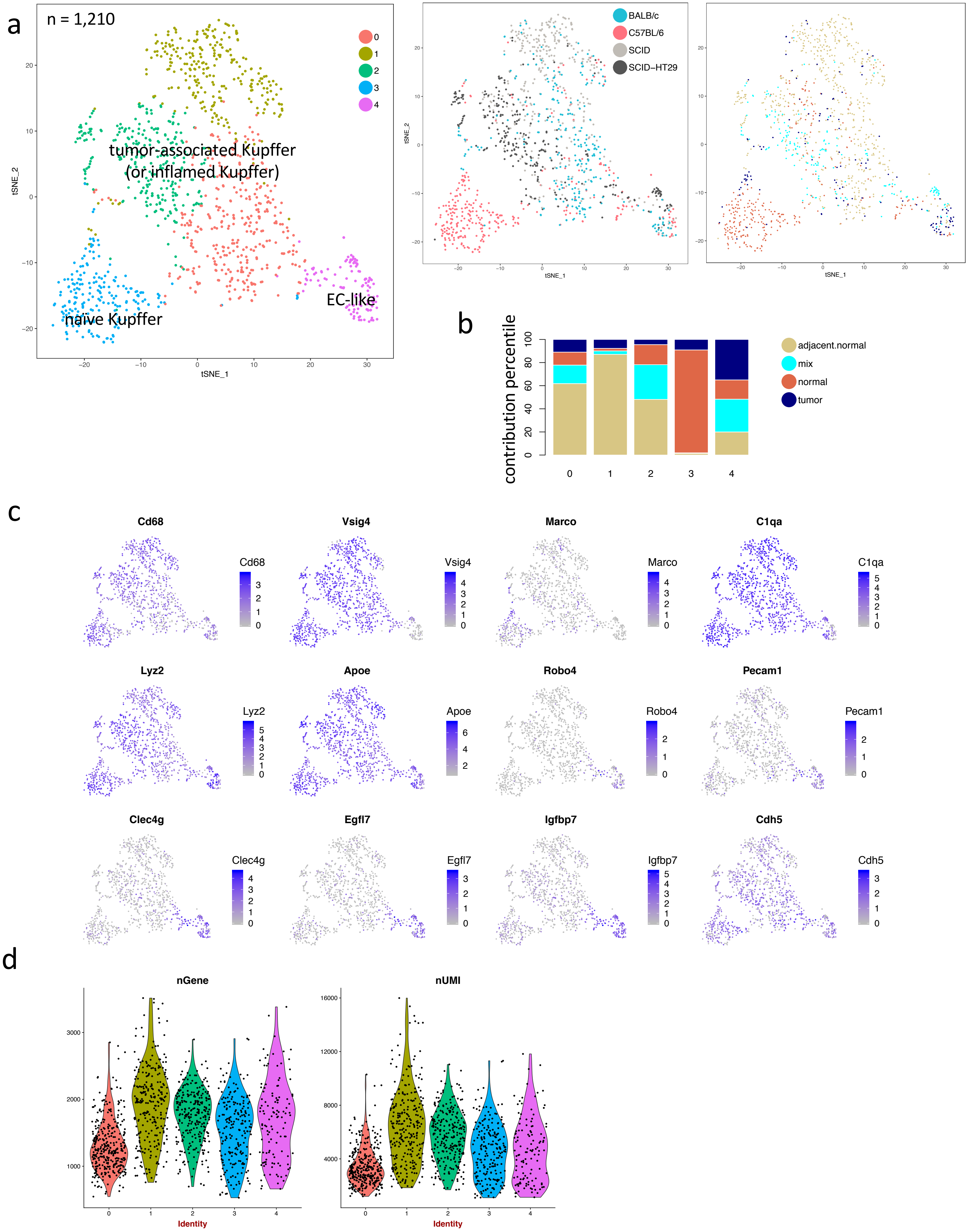

Supplemental Fig. 7

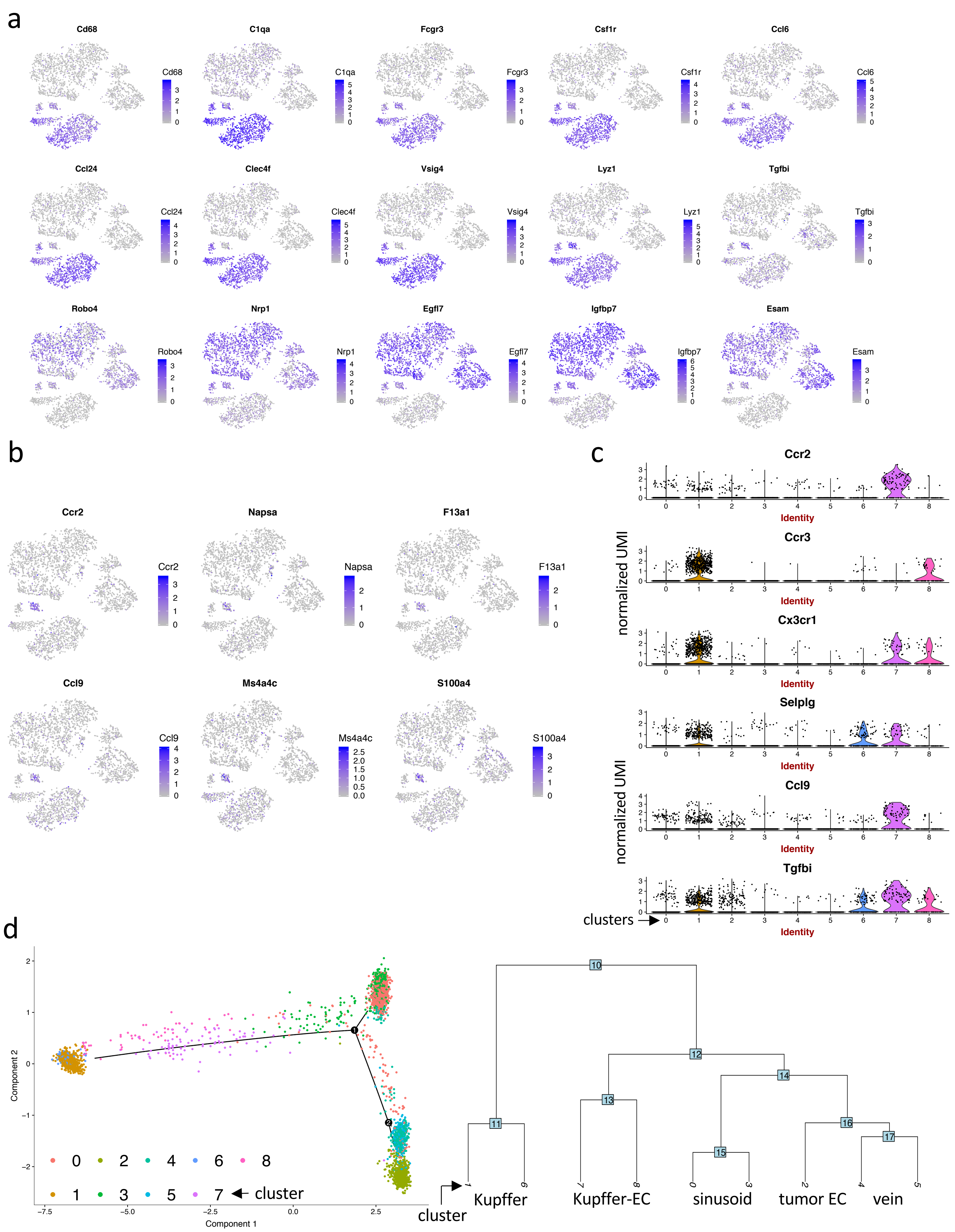

Supplemental Fig. 8

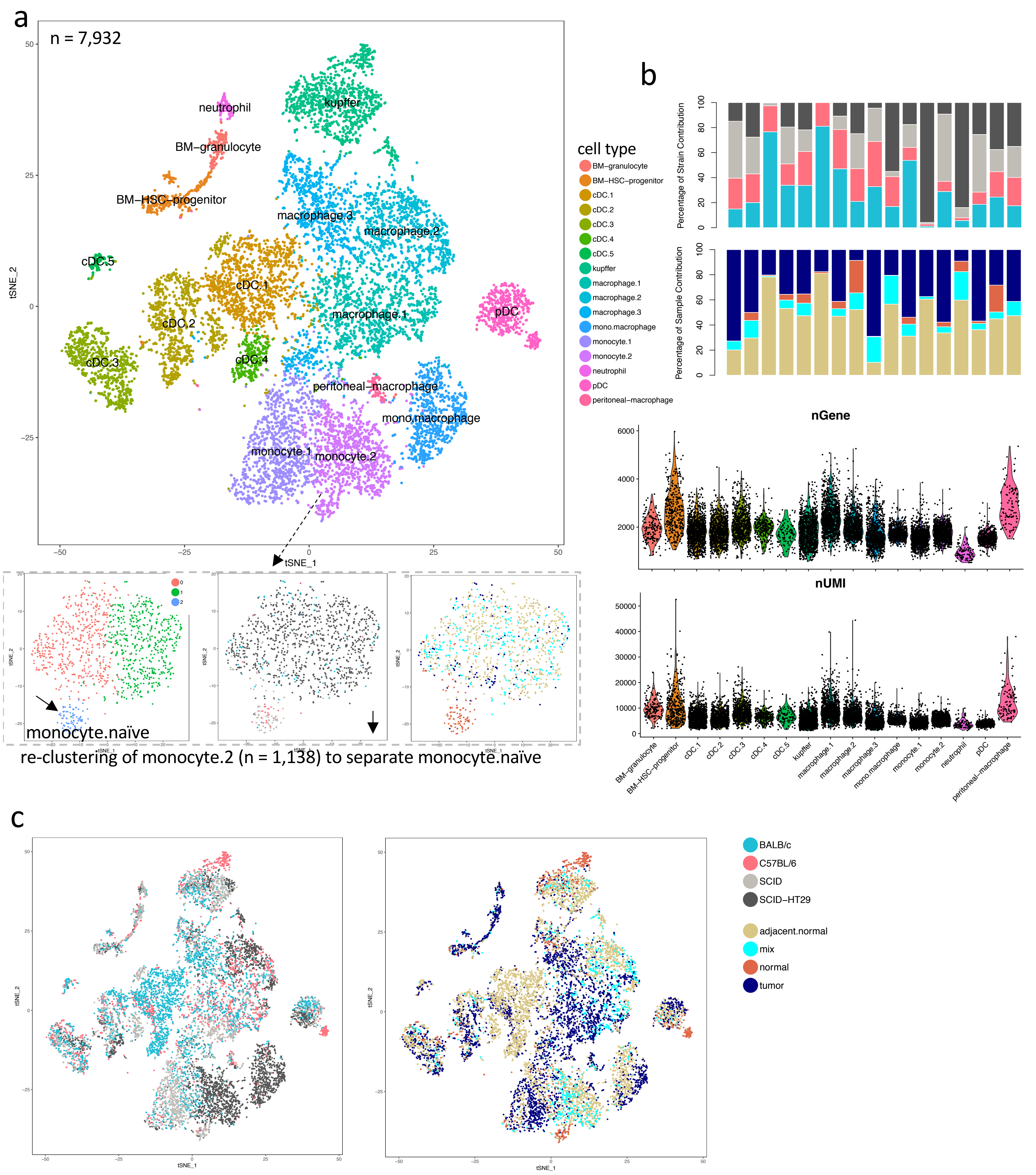

Supplemental Fig. 9

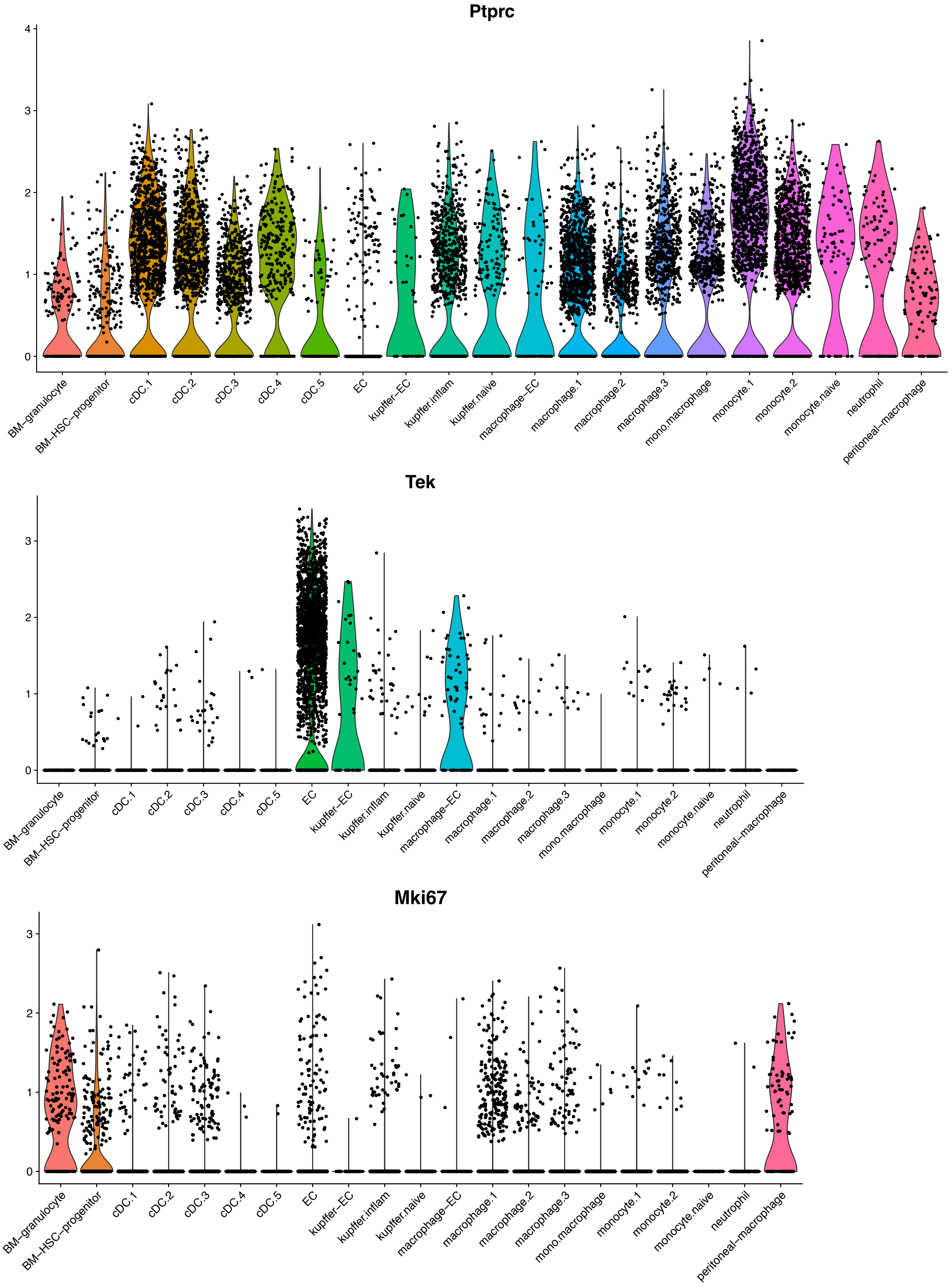

Supplemental Table 5

Sample collections and sequencing batches*

Single cell preparations*	tumor	adjacent normal liver	mix	naïve normal liver
BALB/c - HDD	5	2	1	
C57BL/6 – HDD & naïve	5	1	1	1
SCID – HDD & naïve	3		1	1
SCID-HT29	2	3	1	

*Each preparation is a collection of multiple tumors or tissues and processed separately for single cell sequencing run

Cell counts by strain

strain	BALB/c	C57BL/6	SCID-HDD	SCID-HT29
Cell counts	7,449	8,295	5,213	6,585

Cell counts by sample type

sample type	naïve	adjacent normal	mix	tumor
Cell counts	2,350	8,469	3,062	13,661

Supplemental Figures

Supplemental Fig. 1 (related to Fig. 1) Normal liver EC subpopulations

a. Feature plot (corresponding to Fig. 1a) of selected cell type-specific marker gene expression for annotation of cell clusters. **b.** t-SNE plot of single cells collected from normal liver of C57BL/6 mice, colored by annotated cell type. **c.** Violin plot of total gene and UMI counts by cell type from S. Fig. 1b. **d.** t-SNE plot of single cells collected from normal liver of SCID mice, colored by cluster assignment. Annotation of cluster identity are labeled **e.** Feature plot (corresponding to S. Fig. 1d) showing gene expression of central vein (*Rspo3* and *Wnt9b*), portal vein (*Adgrg6* and *Nrg1*), and sinusoid (*Clec4g* and *Lyve1*) markers in ECs of SCID mice.

Supplemental Fig. 2 (related to Fig. 1) Differences in normal ECs between C57BL/6 and SCID livers

a. Feature plot showing selected gene expression for annotation of clusters identified in combined ECs from normal liver of C57BL/6 and SCID mice (corresponding to Fig. 1d). **b.** Feature plot (corresponding to Fig. 1d) showing expression of differential genes that were highly expressed in the SECs from C57BL/6 mice (*Ly6a, Cd9 and C5ar2*) or SCID mice (*Glo1*). **c.** Expression profile of *Sox17*, *Dll4*, *Efnb2* and *Fabp4* showing zonation patterns across normal liver EC subpopulations (corresponding to line plots in Fig. 1c). **d.** Left, RNAScope confirmation of *Rspo3* as CV marker gene in normal liver of SCID mice. *Pecam1* represented a pan-EC marker as control. Right, cartoon of liver portal triad structure.

Supplemental Fig. 3 (related to Fig. 2) Molecular characterization of HDD-induced intrahepatic tumor ECs

a. Feature plot (corresponding to Fig. 2b t-SNE plot) showing genes (*Col18a1*, *Tmem252*, *Aplnr* and *Vwa1*) specifically expressed and genes (*Angpt2*, *Cav1*, *Inhbb*, *Hbegf*) preferentially expressed by tumor ECs from CD57BL/6 HDD tumors. **b.** t-SNE plot of combined ECs collected from normal liver, dissected tumor, tumor-adjacent normal and undissected tumor-bearing liver (tumor and adjacent normal combined) tissue from SCID mice. Left, cells colored by identified clusters; Right, cells colored by sample type. **c.** Gene and UMI counts across clusters (corresponding to S. Fig. 2b t-SNE plot). **d.** Heatmap of top 15 EC cluster-specific genes. Clusters (corresponding to S. Fig. 2b t-SNE plot) were annotated based on both known marker genes and sample types.

Supplemental Fig. 4 (related to Fig. 4) Characterization of intrahepatic HT-29 tumors and liver tumor tip- and stalk-like cells

a. Gross examination (left) and histological characterization (right) of intrahepatic HT-29 tumors by H&E. **b.** RNAScope of lymphatic marker *Tbx1* in adjacent normal and HT-29 tumor tissues from SCID mice. Arrow indicating tumor cell cluster. **c.** Violin plots showing tip-like cell markers (*Dll4* and *Notch4*) expression in one liver tumor EC subpopulation (tumor.EC1), *Tgfbr3* expression in the other subpopulation (tumor.EC2) and *Kdr* (*Vegfr2*) expression in both subpopulations. **d.** Violin plots showing a few genes identified with differential expression in

tumor ECs between intrahepatically transplanted HT-29 (SCID-HT29) and HDD-induced liver tumors in SCID mice. ‘BALB/c’, ‘C57BL/6’ and ‘SCID’ stand for HDD-derived tumor ECs from those mouse strains.

Supplemental Fig. 5 (related to Fig. 5) Characterization of ECs from various tissues

a. Gene and UMI counts in ECs across clusters as shown in Fig. 5a. Numbers shown on the top are the approximate numbers of cells collected from the respective tissue. **b.** Correlation coefficient scores between subpopulations of normal liver (sinusoid, central vein and portal vein ECs) and normal heart. *, due to limited number of cells captured, liver arteriole and lymphatics populations included ECs from all sample sources (naïve normal, mix, tumor-adjacent normal and tumor). Subcu.tumor.EC is a collection of ECs derived from s.c. tumors including COLO205 and HT-29.

Supplemental Fig. 6 (related to Fig. 6) Identification of a chimeric EC subpopulation in naïve and tumor-bearing liver tissues

a. Cells in the initially defined Kupffer cell cluster subdivided into five subpopulations (numbered 0 to 4). The two main subpopulations were defined as naïve Kupffer (cluster 3) and inflamed (or tumor-associated) Kupffer (cluster 0, 1, 2) based on sample type. A third subpopulation (cluster 4) displayed EC-like characteristics. Left, cells colored by assigned clusters; Right, cells colored by strain (middle) and sample type (right). **b.** Bar plot showing sample type distribution in percentage across all 5 clusters. **c.** Expression of marker genes used to annotate each cluster, including cluster 4 expressing both EC and myeloid cell markers. **d.** Gene and UMI counts in Kupffer cell clusters.

Supplemental Fig. 7 (related to Fig. 6) Characterization of chimeric ECs

a. Feature plot (corresponding to Fig. 6a) of additional marker genes, showing the chimeric EC property in cluster 7 and 8. **b.** Feature plot (corresponding to Fig. 6a) showing expression of cluster 7-specific genes. Evidence of unique genes expressed by cells in cluster 7 suggested that this subpopulation was unlikely derived from doublet or phagocytosis. **c.** Violin plot of selected cytokines and receptors expression in different clusters (corresponding to Fig. 6a). **d.** Trajectory (left) and Cluster Tree (right) analyses on Kupffer cells, ECs, and chimeric ECs (clusters labeled corresponding to Fig. 6a).

Supplemental Fig. 8. (related to Fig. 6) Identification of cell populations and subpopulations in all liver myeloid cells

a. Identification of subpopulations of all myeloid cells by merging all samples and performing re-clustering, with some subpopulations introduced by the presence of tumor or strain variation. An example on monocyte.2 zoom-in showcased how three subpopulations of monocytes (monocyte.naïve, monocyte.inflam1 and monocyte.inflam2) were annotated. Marker genes used to annotate each subpopulation are listed in supplemental table 4. **b.** Cell distribution by strain and sample type (top) as well as gene and UMI counts across myeloid subpopulations (bottom). **c.** The same t-SNE plot (in S. 8A) colored by strain and sample type.

Supplemental Fig. 9 (related to Fig. 6)

Violin plots showing expression of *Ptprc* (*Cd45*, a leukocyte marker), *Tek* (alias *Tie2*, an EC marker) and *Mki67* (a marker for cell proliferation) in cell populations shown in Fig. 6e.

Supplemental Tables

S. T1. Normal liver EC subpopulation marker genes and transcriptome difference between C57BL/6 and SCID liver EC.

S. T2. Tumor EC cluster-specific genes and tumor-induced gene expression changes.

S. T3. Differentially expressed genes in tumor ECs between liver and s.c.

S. T4. Top marker genes used for annotation of major myeloid subpopulations.

S. T5. Summary on sample collection and sequencing runs as well as cell counts.
